# Interventions for opioid use disorder during the first year postpartum: A systematic review

**DOI:** 10.1002/pmf2.70256

**Published:** 2026-02-09

**Authors:** Charlotte B. McCarley, Pranaya Chilukuri, Juliann Wang, Brian E. Brocato, Carolyn M. Webster, Brian Casey, Rachel G. Sinkey

**Affiliations:** ^1^ Department of Obstetrics and Gynecology University of Alabama at Birmingham Birmingham Alabama USA; ^2^ Center for Research in Women's Health University of Alabama at Birmingham Birmingham Alabama USA; ^3^ Department of Obstetrics and Gynecology University of Kentucky Lexington Kentucky USA; ^4^ Heersink School of Medicine University of Alabama at Birmingham Birmingham Alabama USA; ^5^ Present address: Department of Obstetrics and Gynecology University of Tennessee Health Sciences Center College of Medicine—Knoxville Knoxville Tennessee USA; ^6^ Present address: Department of Obstetrics and Gynecology Vanderbilt University Medical Center Nashville Tennessee USA; ^7^ Present address: Department of Family and Social Medicine Albert Einstein College of Medicine Bronx New York USA; ^8^ Present address: Department of Obstetrics, Gynecology, and Reproductive Sciences West Virginia Medicine Morgantown West Virginia USA

**Keywords:** postpartum, pregnancy, substance use disorder

## Abstract

**Objective:**

To provide a qualitative summary of published interventions for postpartum opioid abstinence and reduction in non‐prescribed opioid use in patients with opioid use disorder (OUD).

**Materials and Methods:**

PubMed, CINAHL, PsychINFO, and EMBASE were searched from inception to January 11, 2023. Intervention trials with ≥5 subjects were included if they reported an intervention aimed at opioid abstinence and reduction in non‐prescribed opioid use within the first year postpartum in patients with OUD. Studies without a comparison group or intervention not continued into the postpartum period were excluded. Two reviewers independently screened citations and summarized findings from eligible studies using Covidence®. Risk of bias was assessed with Cochrane Risk of Bias or Newcastle‐Ottawa Scale as appropriate.

**Results:**

Of the 812 articles screened, 14 were assessed for full‐text eligibility and three were included for full‐text review. Trials employed a variety of strategies to reduce opioid use including employment for continued abstinence (*n* = 40), a comprehensive clinical care model (*n* = 213), and motivational‐enhancement therapy (*n* = 60). Dropout rates were high in two studies, ranging from 22% to 60%, with no dropout noted in the third study. Risk of bias varied between studies.

**Conclusions:**

Despite the large contribution of OUD to maternal mortality, only three intervention studies assessing strategies for postpartum opioid abstinence and reduction in non‐prescribed opioid use in patients with OUD were identified. Existing studies were heterogenous and showed limited success in postpartum opioid abstinence and reduction in non‐prescribed opioid use among patients with OUD. This underscores the pressing need to address this significant public health concern and contributor to maternal mortality.

## 1 INTRODUCTION

Opioid use disorder (OUD) in pregnancy has significantly increased in recent years, mirroring the nationwide opioid epidemic [1]. According to the Centers for Disease Control and Prevention, the prevalence of OUD in pregnancy increased fourfold from 1999 to 2014 (1.5/1000 to 6.5/1000 delivery hospitalizations, respectively) with continued increase noted in 2017 (8.2/1000 delivery hospitalizations) [2, 3]. Along with the rise in opioid use during pregnancy, maternal substance use disorder (SUD) has been identified as a leading contributor to maternal mortality [4, 5]. Multiple states have reported overdose as a leading cause of pregnancy‐associated deaths, including Alabama, Virginia, Tennessee, and Maryland, with Maryland reporting that substance use and unintentional overdose contributed to 38% of pregnancy‐associated deaths each year from 2010 to 2017 [4, 5, 6, 7]. On a national scale, late postpartum deaths (43 days to 1 year postpartum) nearly doubled from 3.1 per 100,000 live births from January to June 2018 to 6.1 per 100,000 live births from July to December 2021, largely driven by SUD [8].

The postpartum period is a particularly high‐risk time for recurrence of use and treatment dropout, and contributes to increasing rates of SUD‐related maternal death [9, 10, 11]. The use of medications for OUD (MOUD) and behavioral‐based healthcare for OUD during pregnancy and postpartum have consistently been shown to decrease overdose risk and improve both maternal and child outcomes [12]. However, only 44% of patients receiving treatment for OUD during pregnancy continue treatment at 6 months postpartum despite recommendations from the American College of Obstetricians and Gynecologists (ACOG) and others that treatment, specifically MOUD, continue after delivery [1, 8, 9, 10, 11]. This is likely multifactorial: due to the scarcity of treatment programs that extend to the late postpartum period; the transition of treatment providers from obstetric to primary care providers at 6–12 weeks postpartum; the loss of postpartum health insurance coverage, particularly in those with public health insurance; and existing co‐morbid mental health disorders.

Maternal mortality review committees have recommended policy changes to increase access to both prenatal care and MOUD in pregnant and postpartum patients with SUD [4]. Although advances have been made for antenatal treatment, gaps remain in the transition of care to the postpartum period, which has historically lacked high‐quality (Level I) evidence to guide effective interventions for treatment of SUD/OUD in the postpartum period. Therefore, our objective was to conduct a systematic review to provide a qualitative summary of published interventions for postpartum opioid abstinence and reduction in non‐prescribed opioid use in patients with OUD.

## 2 MATERIALS AND METHODS

The systematic review protocol was developed with multidisciplinary input from maternal‐fetal medicine subspecialists and a psychologist specializing in maternal mental health, with critical support from a librarian experienced in the conduct of systematic reviews. PROSPERO registration (CRD42023405688) occurred on March 17, 2023, before the results were screened or data extraction had begun. The study was conducted in accordance with the PRISMA (Preferred Items for Reporting in Systematic Reviews and Meta‐Analyses) Guidelines [13].

Intervention studies with primary or secondary outcomes aimed at postpartum opioid abstinence and reduction in non‐prescribed opioid use among patients with OUD were included. The intervention must have started during or extended into the postpartum period, but did not have to last the entire 12 months postpartum. All intervention types including MOUD, psychotherapy, or behavioral health interventions were included. Comparative studies (randomized controlled trials [RCT] or prospective or retrospective non‐randomized comparative studies) with ≥5 participants who used opioids for nonmedical reasons or who had a diagnosis of OUD and that were published in English in a peer‐reviewed journal were included. Studies were excluded if they did not include human subjects, a comparator group, or in which interventions did not extend to the postpartum period. Studies in which the patients used other substances in addition to opioids were included.

Literature searches were conducted in MEDLINE, ClinicalTrials.gov, PubMed, CINAHL, PsychINFO, and EMBASE from inception through January 11, 2023 by a librarian who specializes in systematic reviews at our institution.

The search included terms for OUD, behavioral therapies, and postpartum period; the full search strategy can be found in Appendix S1. Covidence® was used to import and manage citations [14].

Each abstract was screened independently by two reviewers (P.C. and J.W.) using the online Covidence® reference management system [14]. Next, potentially relevant full‐text articles were screened by two reviewers (P.C. and J.W.). Disagreements were adjudicated by a third reviewer (C.B.M.) [14].

Baseline characteristics including participant age, race, and non‐prescription drug use were collected. The primary outcome of interest was postpartum opioid abstinence and reduction in non‐prescribed opioid use defined as no or decreased opioid use after discharge from the delivery‐associated hospitalization. Secondary outcomes of interest included maternal mortality from opioid overdose within the first year postpartum, opioid overdose defined as requiring treatment for overdose, treatment adherence, uptake of MOUD, co‐morbid mental health diagnoses, feasibility or acceptability, hospital and/or facility admission, hospital/facility length of stay, neonatal opioid abstinence syndrome, and neonatal mortality.

Data on study design, baseline characteristics, interventions, outcomes, and results were extracted and confirmed and/or refuted by at least one other reviewer. Discrepancies were resolved by the senior author.

Risk of bias of each study was assessed using the Cochrane Risk of Bias tool 2.0 for RCTs and the Newcastle‐Ottawa Risk of Bias tool for non‐randomized comparative studies [15, 16]. Specifically, each study was evaluated for selection, performance, detection, attrition, and reporting according to the Cochrane Risk of Bias tool and other biases or selection, comparison, and outcome bias according to the Newcastle‐Ottawa Risk of Bias tool [15, 16]. Articles were deemed to have low, high, or unclear risk of bias based on this evaluation [16].

A quantitative meta‐analysis was not planned a priori as the primary objective of the review was to synthesize the available literature as part of a broader initiative to improve postpartum care for patients with SUD.

## 3 RESULTS

**FIGURE 1:**
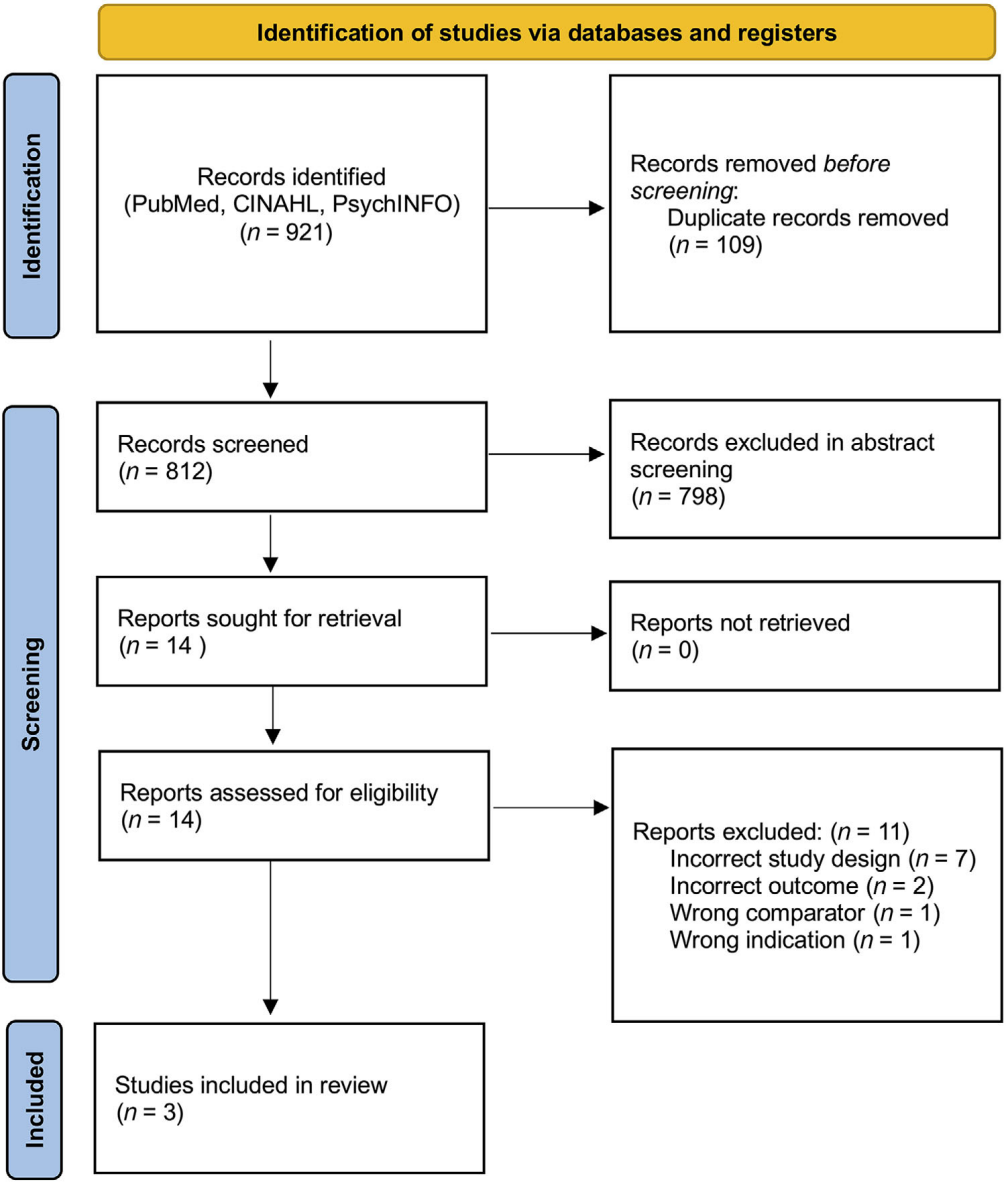
Literature flow diagram.

The literature search yielded 812 citations, of which 14 were retrieved for full‐text review [12, 17, 18, 19, 20, 21, 22, 23, 24, 25, 26, 27, 28, 29]. Ultimately, based on our search criteria, three studies met the inclusion criteria (Figure 1) [26, 27, 28]. Most full texts were rejected because they did not include the correct study design (*n* = 7) [18, 19, 20, 21, 22, 24, 29]. The 11 studies that were retrieved for full review, but ultimately did not meet criteria to be included in the study, are summarized in Table S1. Each included study evaluated a different intervention, including a therapeutic workplace, comprehensive perinatal care, and motivational‐enhancement therapy [26, 27, 28].

Table 1 summarizes the study design and outcomes of the three included studies. Silverman et al. conducted an RCT in the United States assessing a therapeutic workplace that provided employment contingent on daily negative urine drug screens and included time‐based incentives for opioid abstinence [26]. The therapeutic workplace included education and training for data entry jobs. Participants were invited to attend the workplace 3 h/day from Monday through Friday and received a base pay rate that increased with consecutive abstinence. Included participants were pregnant or postpartum aged 18–50 years who were unemployed and on methadone maintenance. Exclusion criteria included any condition that may disrupt their ability to work or provide informed consent. There were 40 participants (20 therapeutic workplace, 20 usual care) who were followed for 6 months and assessed for opioid abstinence. At 6 months, there was a non‐significant increase in opioid abstinence in the therapeutic workplace compared to usual care group (52% vs. 33%, *p* = 0.06). Additionally, retention in the program was low with 12 (60%) usual care and 9 (45%) therapeutic workplace participants discharged from the program before the end of the study, most commonly for failing to comply with program rules.

**TABLE 1:** Study design, sample characteristics, and results.

Study, country	Design, years, sample size	Intervention	Primary outcome	Inclusion and exclusion criteria	Study results	Treatment adherence	Secondary outcomes^a^	Risk of bias
Silverman et al. 2001 [26], United States	RCT 1996–1998 *n* = 40	Therapeutic workplace	Opioid abstinence	*Inclusion criteria*: Pregnant or postpartum patients aged 18–50 years, unemployed, on methadone maintenance with ≥1 UDS positive for opioids or cocaine within 6 weeks of enrollment *Exclusion criteria*: At risk for suicide or had psychiatric disorder that might disrupt ability to work or provide informed consent	Increase in opioid abstinence after 6 months (UC 33% vs. TW 52%, *p* = 0.060)	8/20 (40%) maintained high attendance rates	Feasibility: Described need for self‐sustaining funding for the workplace outside of the experimental setting	Low
Wright et al. 2012 [27], United States	PC 2007–2010 *n* = 213	Comprehensive perinatal care	Program effectiveness as determined by four domains (birth outcomes, drug use, custody, repeat pregnancy)	*Inclusion criteria*: Pregnant and postpartum patients with SUD *Exclusion criteria*: None	Abstinence at delivery (93/97, 96%) Recurrence of use to heavy substance usage at 6 months postpartum (13%)	Not reported		High
Esmaeilinejad Hasaroeih et al. 2022 [28], Iran	RCT 2019 *n* = 60	Motivational‐enhancement therapy	Effect of therapy on substance use patterns	*Inclusion criteria*: Postpartum patients aged 15–45 years with SUD *Exclusion criteria*: Absence from one session, committing a crime, using other counseling services	Reduction in the *number* of opium (*p* = 0.04) and methadone (*p* = 0.03) users. Reduction in the *amount* of opium (*p* = 0.04) and methadone (*p* = 0.02).	100%		Low

Abbreviations: PC, prospective cohort; RCT, randomized controlled trial; SUD, substance use disorder; TW, therapeutic workplace; UC, usual care; UDS, urine drug screen.

^a^ Secondary outcomes included maternal mortality from opioid overdose within the first year postpartum, opioid overdose defined as requiring treatment for overdose, treatment adherence, uptake of medications for opioid use disorder, co‐morbid mental health diagnoses, feasibility or acceptability, hospital and/or facility admission, hospital/facility length of stay, neonatal opioid abstinence syndrome, and neonatal mortality. If not listed, the outcome was not applicable or not reported by the authors.

Wright et al. conducted a prospective cohort study in the United States of 213 patients with active drug use or who were recently abstinent and at risk of recurrence of use who participated in a comprehensive perinatal addiction treatment center with a harm‐reduction model compared to those who received usual care [27]. As part of this model, patients were seen in an off‐site clinic providing a “home‐like setting” which provided childcare, food, and transportation. Available services included Obstetrics, Obstetrical Ultrasound, Addiction Psychiatry, Addiction Medicine, Social Services, and several group classes including one for recurrence of use prevention. The success of the comprehensive perinatal care model was assessed via four domains with drug use being one of the domains. Most of the population used methamphetamines (184/213, 86%) with only 4% (9/213) of participants using opioids. Of the 97 patients who delivered during the study period, 93.7% (90/97) were abstinent at delivery; however, 22% were lost to follow up and 13% experienced recurrence of use to heavy substance use at 6 months postpartum.

The third study, by Esmaeilinejad Hasaroeih et al., was an RCT conducted in Iran that evaluated motivational‐enhancement therapy versus usual care in 60 postpartum patients with SUD [28]. Participants randomized to the intervention arm attended four individual motivational‐enhancement therapy sessions and had four follow‐up telephone visits. Each session focused on a separate component of the motivational‐enhancement therapy protocol including understanding implications of drug use, feedback on consequences of drug use, promoting healthy lifestyle changes, strategies for implementing those changes, and identifying and overcoming barriers to sustained abstinence. The most commonly used drugs were opium (33/60, 55%) and non‐prescribed methadone (14/60, 23%). There was a significant reduction in the number of opium users after the intervention (15 [50%] vs. 7 [23.3%]; *p* = 0.008). However, there was an increase in methadone use after the intervention (9 [30%] vs. 15 [50%]; *p* = 0.03), suggesting that some patients started on prescribed methadone after the intervention. There was a decrease in the overall amount of opium (2.88±1.02 g vs. 1.72±1.22 g, *p* = 0.01) and methadone (28.66 ± 16.5 mg vs. 20.66 ± 5.5 mg, *p* = 0.13) used per day after the intervention.

**FIGURE 2:**
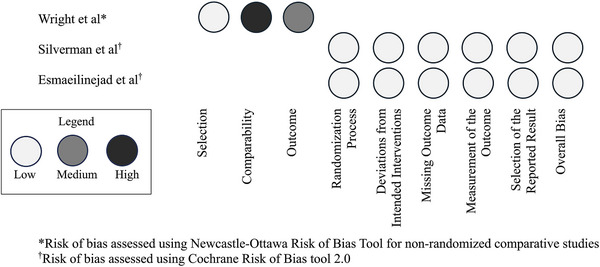
Evaluation of risk of bias.

The risk of bias for each RCT was determined to be low by the Cochrane Risk of Bias tool (Figure 2) [16, 26, 28, 30]. Risk of bias for the study by Wright et al. [27] was determined to be high by the Newcastle‐Ottawa Scale secondary to low scores in the comparability and outcome domains, indicating that there was limited control for confounders between the two groups and limited follow‐up [15].

## 4 DISCUSSION

Our systematic review only identified three intervention studies aimed at assessing strategies for opioid abstinence and reduction in non‐prescribed opioid use in postpartum patients with OUD. The studies were heterogenous both in design and results, varying widely in the intervention tested, country in which the study was conducted, types of patients included (participants with OUD and those with polysubstance use disorder), and success of the intervention. Each study had a relatively small sample size and did not exclusively study OUD since many of the patients used multiple substances. Additionally, none of the studies included MOUD (i.e., buprenorphine or methadone) as a postpartum intervention strategy for decreasing non‐prescription postpartum opioid use.

The study by Esmaeilinejad Hasaroeih et al. [28] specifically examined the postpartum period and was the only study whose intervention was associated with reduced postpartum opioid use. The investigators employed motivational‐enhancement therapy for eight patient interactions in which information was shared regarding substance use patterns and its physical and psychological effects, feedback on those consequences, developing a plan to change patterns, and identifying methods for maintaining that change [28]. This technique is similar to motivational interviewing that has been used outside of the perinatal period to prompt motivation for behavioral change and adherence to treatment [31]. Notably, this study was conducted in Iran, which limits the generalizability to patients in the United States given significant cultural and healthcare system differences between the two populations. The success of this strategy, which used psychiatric care in the postpartum period, highlights the importance of sustained access to SUD treatment, which is unfortunately only available to <10% of all people with SUD in the United States [32]. Given the potential efficacy of motivational‐enhancement therapy to improve outcomes in postpartum patients with SUD, a multicenter study including a cost‐effectiveness analysis would provide additional evidence for or against its integration in clinical practice.

Rates of dropout and loss to follow up were high in two of our included studies, ranging from 22% to 60% [26, 27]. This is not surprising considering that approximately 40% of all patients do not attend their postpartum visit for reasons such as feeling well, childcare needs, loss of insurance coverage, or other social demands [33, 34]. In addition, greater than 50% of patients with OUD face perceived stigma in healthcare and associated psychological distress in the prenatal and postpartum periods, which may further limit their engagement in follow‐up [35].

A key strength of this review was only including studies with a comparator group so that the interventions were compared to the standard of care. Several observational studies on this topic have been published, but without a comparator, it is not possible to assess potential improvement offered by the intervention. Additionally, the search was conducted by a specialized librarian with advanced training, enhancing the likelihood that pertinent articles were captured in the search. There are several limitations to this systematic review, however, including that each study had a different methodology, assessment of outcomes varied across studies, the duration of treatment, time frame during which outcomes were ascertained, and the country of origin differed, all of which may affect the results and their interpretation. Furthermore, studies did not necessarily include opioid abstinence and reduction in non‐prescribed opioid use as the primary outcome and information regarding our secondary outcomes was extremely limited.

## 5 CONCLUSION

While few intervention trials met the inclusion criteria for this systematic review, motivational‐enhancement therapy was identified as a possible strategy for opioid abstinence and reduction in non‐prescribed opioid use postpartum. It is essential that we invest in clinical trials that can provide high‐level evidence for strategies similar to those presented in this systematic review to allow for allocation of funding in support of treatment of OUD in the postpartum period. Proper design and evaluation of timely interventions are costly but important and will only be made possible by increasing federal and other funding opportunities. The rising contribution of OUD to preventable maternal deaths in the United States and the paucity of interventions to treat postpartum patients with OUD as demonstrated by this systematic review highlights the urgency to identify successful interventions to treat this high‐risk group of patients [4, 5, 6, 7, 8, 36].

## ACKNOWLDEGMENTS

The authors acknowledge the work of Jill Deaver, MA, MLIS. Artificial Intelligence was used to develop the Supporting Information table that was reviewed for accuracy and edited by the primary author.

## CONFLICT OF INTEREST STATEMENT

The authors declare no conflicts of interest.

## FUNDING INFORMATION

The authors received no specific funding for this work.

## Supporting information



Supporting information

## Data Availability

Data sharing not applicable to this article as no datasets were generated or analyzed during the current study.
