# Supplementary material for: Interventions for opioid use disorder during the first year postpartum: A systematic review
Source: Pregnancy (Hoboken). 2026 Feb 9;2(2):e70256. doi: 10.1002/pmf2.70256 (PMC13344366; doi:10.1002/pmf2.70256)
Supplement: Supplementary file 1 [file PMF2-2-e70256-s001.docx]

**Supplemental Appendix 1. Methods**

**Database Search Strategies**

**Medline and ClinicalTrials.gov via PubMed**

("Opioid Epidemic"[Mesh]

OR "Opioid-Related Disorders"[Mesh]

OR opiate*[tiab]

OR opioid*[tiab])

AND

(("Opiate Substitution Treatment"[Mesh]

OR substitution-treatment*[tiab])

OR ("Behavior and Behavior Mechanisms"[Mesh]

OR "Behavioral Disciplines and Activities"[Mesh]

OR "Behavior Therapy"[Mesh] OR behavior*[tiab] OR psycholog*[tiab]))

AND

(postpartum[tiab]

OR "Postpartum Period"[Mesh])

AND

("Behavior and Behavior Mechanisms"[Mesh]

OR "Behavioral Disciplines and Activities"[Mesh]

OR "Behavior Therapy"[Mesh] OR behavior*[tiab]

OR psycholog*[tiab])

**CINAHL**

(opiate*

OR opioid*

OR MH "Analgesics, Opioid+"

OR MH "Opioid Epidemic"

OR MH "Opium+" OR MH "Narcotics )

AND

( postpartum OR postnatal

OR MH "Postnatal Period" )

AND

behavior*

Limiters: Academic Journals 558

**PsycINFO**

(opiate*

OR opioid*

OR "Opioid Epidemic" )

AND

( postpartum

OR postnatal

OR MH "Postnatal Period" )

Limiters: English, humans, journal article

**Embase**

('algopan'

OR 'biopon'

OR 'cofapon'

OR 'dropizol'

OR 'laudanon'

OR 'laudanum'

OR 'laudopan'

OR 'nepenthe'

OR 'omnopon'

OR 'opial'

OR 'opiate alkaloid'

OR 'opiate alkaloids'

OR 'opioid'

OR 'opium'

OR 'opium alkaloid'

OR 'opium alkaloids'

OR 'opium derivative'

OR 'opium poppy'

OR 'opium tincture'

OR 'opon'

OR 'oposal'

OR 'pantopon'

OR 'pantopone'

OR 'papaveretum'

OR 'pavon'

OR 'tetrapon'

OR 'opiate addict'

OR 'opiate alkaloid addict'

OR 'opiate alkaloid dependency'

OR 'opiate alkaloids addict'

OR 'opiate crisis'

OR 'opiate dependence'

OR 'opiate dependency'

OR 'opiate epidemic'

OR 'opioid addict'

OR 'opioid addiction'

OR 'opioid crisis'

OR 'opioid dependence'

OR 'opioid dependency'

OR 'opioid epidemic'

OR 'opioid-related disorders'

OR 'opioids addict'

OR 'opioids addiction'

OR 'opioids crisis'

OR 'opioids dependence'

OR 'opioids dependency'

OR 'opioids epidemic'

OR 'opium addict'

OR 'opium addiction'

OR 'opium alkaloid addict'

OR 'opium alkaloid addiction'

OR 'opium dependence'

OR 'opium dependency'

OR 'opium epidemic')

AND

('life, perinatal'

OR 'perinatal life'

OR 'peripartum period'

OR 'postnatal period')

| **Supplemental Table 1. Summary of Studies Included in Full Text Review** | | | |
| --- | --- | --- | --- |
| **First author** | **Year** | **Country of Origin** | **Summary** |
| Crane^12^ | 2019 | United States | Describes a statewide quality improvement initiative to improve maternal and infant health outcomes and family stability among pregnant individuals with opioid use disorder through coordinated, standardized care. |
| Jones^17^ | 2020 | United States | Reports a quality improvement initiative focused on reducing inpatient opioid use among all postpartum patients by implementing multimodal, opioid-sparing pain management strategies. |
| O’Connor^18^ | 2018 | United States | Retrospective cohort study aimed at identifying predictors of treatment retention among postpartum individuals prescribed buprenorphine during pregnancy. |
| Cochran^19^ | 2018 | United States | A 1-group repeated measures pilot study aimed evaluating a multidisciplinary, patient-centered treatment model for pregnant women with opioid use disorder. |
| Cleary (EM)^20^ | 2020 | United States | Prospective, open-label cohort study which describes clinical use of extended-release subcutaneous buprenorphine among pregnant and postpartum individuals, focusing on feasibility and early clinical observations. |
| Wilder^21^ | 2017 | United States | Retrospective cohort study examining the relationship between methadone dose ≥ 60mg per day and treatment retention among pregnant and postpartum women with opioid use disorder. |
| Mittal^22^ | 2017 | United States | Case series of 16 pregnancies with individuals prescribed buprenorphine which evaluates the feasibility of a collaborative care model for treating opioid use disorder during pregnancy. |
| Ahrens^23^ | 2022 | United States | Retrospective cohort evaluating postpartum health care utilization among pregnant persons with opioid use disorder receiving medication for addiction treatment. It demonstrated decreased emergency health care utilization in those receiving medications for opioid use disorder |
| Cleary (BJ)^24^ | 2013 | Ireland | Prospective cohort study investigating methadone dosing patterns and neonatal abstinence syndrome between those with dose decreases and those with steady or increasing doses. |
| Metz^25^ | 2014 | Austria | Describes demographic characteristics, health status, and quality of life among opioid-dependent pregnant women in Austria. |
| Aklin^29^ | 2014 | United States | Reports eight-year outcomes of a therapeutic workplace social business intervention for long-term treatment of substance use disorder and unemployment, demonstrating sustained improvements in employment and substance use outcomes. |
